# Retrospective clinical trial experimentally validates glioblastoma genome-wide pattern of DNA copy-number alterations predictor of survival

**DOI:** 10.1063/1.5142559

**Published:** 2020-05-15

**Authors:** Sri Priya Ponnapalli, Matthew W. Bradley, Karen Devine, Jay Bowen, Sara E. Coppens, Kristen M. Leraas, Brett A. Milash, Fuqiang Li, Huijuan Luo, Shi Qiu, Kui Wu, Huanming Yang, Carl T. Wittwer, Cheryl A. Palmer, Randy L. Jensen, Julie M. Gastier-Foster, Heidi A. Hanson, Jill S. Barnholtz-Sloan, Orly Alter

**Affiliations:** 1 Scientific Computing and Imaging Institute, University of Utah, Salt Lake City, Utah 84112, USA; 2 Department of Bioengineering, University of Utah, Salt Lake City, Utah 84112, USA; 3 Department of Population and Quantitative Health Sciences, Case Western Reserve University School of Medicine, Cleveland, Ohio 44106, USA; 4 The Research Institute at Nationwide Children's Hospital, Columbus, Ohio 43205, USA; 5 Center for High-Performance Computing, University of Utah, Salt Lake City, Utah 84112, USA; 6 Beijing Genomics Institute (BGI) -Shenzhen, Shenzhen, Guangdong 518083, China; 7 BGI-Americas, Cambridge, Massachusetts 02142, USA; 8 China National GeneBank, Shenzhen, Guangdong 518120, China; 9 Department of Pathology, University of Utah, Salt Lake City, Utah 84112, USA; 10 Huntsman Cancer Institute, University of Utah, Salt Lake City, Utah 84112, USA; 11 Department of Neurosurgery, University of Utah, Salt Lake City, Utah 84112, USA; 12 Department of Surgery, University of Utah, Salt Lake City, Utah 84112, USA; 13 Utah Population Database, University of Utah, Salt Lake City, Utah 84112, USA; 14 Department of Human Genetics, University of Utah, Salt Lake City, Utah 84112, USA

## Abstract

Modeling of genomic profiles from the Cancer Genome Atlas (TCGA) by using recently developed mathematical frameworks has associated a genome-wide pattern of DNA copy-number alterations with a shorter, roughly one-year, median survival time in glioblastoma (GBM) patients. Here, to experimentally test this relationship, we whole-genome sequenced DNA from tumor samples of patients. We show that the patients represent the U.S. adult GBM population in terms of most normal and disease phenotypes. Intratumor heterogeneity affects ≈11% and profiling technology and reference human genome specifics affect <1% of the classifications of the tumors by the pattern, where experimental batch effects normally reduce the reproducibility, i.e., precision, of classifications based upon between one to a few hundred genomic loci by >30%. With a 2.25-year Kaplan–Meier median survival difference, a 3.5 univariate Cox hazard ratio, and a 0.78 concordance index, i.e., accuracy, the pattern predicts survival better than and independent of age at diagnosis, which has been the best indicator since 1950. The prognostic classification by the pattern may, therefore, help to manage GBM pseudoprogression. The diagnostic classification may help drugs progress to regulatory approval. The therapeutic predictions, of previously unrecognized targets that are correlated with survival, may lead to new drugs. Other methods missed this relationship in the roughly 3B-nucleotide genomes of the small, order of magnitude of 100, patient cohorts, e.g., from TCGA. Previous attempts to associate GBM genotypes with patient phenotypes were unsuccessful. This is a proof of principle that the frameworks are uniquely suitable for discovering clinically actionable genotype–phenotype relationships.

## INTRODUCTION

The prognostics, diagnostics, and therapeutics of glioblastoma (GBM), which is the most prevalent as well as most aggressive brain cancer in adults, have remained largely unchanged for decades. Only one drug, i.e., the alkylating agent temozolomide, has progressed from clinical trials to standard of care since 1980, modestly improving the median life expectancy of patients treated by surgical resection and radiation to roughly 15 months.^1–3^ A biomarker of response to earlier alkalyting agents in different types of cancer, i.e., the methylation of the promoter of the gene O^6^-methylguanine-DNA methyltransferase (*MGMT*), is being used today also to predict GBM response to temozolomide and indicate survival.^4,5^ Only two additional biomarkers have progressed from omic studies to GBM standard of care, both of which have already been used as indicators of survival in other types of cancer, i.e., the mutation of the gene isocitrate dehydrogenase 1 (*IDH1*) and, most recently, the mutation of the promoter of the gene telomerase reverse transcriptase (*TERT*), which is correlated with the messenger RNA (mRNA) expression of the gene.^6–9^ Efforts to conclusively associate outcome, e.g., survival, with GBM-specific mRNA expression of between a few to a few hundred genes, have been unsuccessful, and have not been translated into clinical use.^10–12^ Despite advances in profiling technologies, including an authorization by the Food and Drug Administration (FDA) for non-disease-specific applications of next-generation sequencing,^13^ and the growing numbers of publicly available (gen)omic datasets, age at diagnosis has remained the best indicator of GBM survival in clinical practice since 1950.^14–16^

At the same time, recurrent abnormal numbers and sizes of chromosomes, which have been recognized as a hallmark of cancer since 1914,^17^ have been observed in GBM for decades without being translated to the clinic. Following the World Health Organization (WHO) recommendations, other types of cancer are classified and treated based upon recurrent changes in chromosome and focal DNA copy numbers. For example, the observation of the so-called “Philadelphia chromosome” in myeloid leukemia led to a drug that converts most cases of the blood cancer from a fatal disease into a manageable chronic condition.^18–20^ In another example, co-deletion of the short arm of chromosome 1, i.e., 1p, and the long arm of chromosome 19, i.e., 19q, is used to distinguish oligodendroglioma from other brain cancers, including GBM, i.e., grade IV astrocytoma. Repeated previous attempts, however, to associate GBM-specific copy-number genotypes with clinical phenotypes, e.g., survival, were unsuccessful.^21–23^

**FIG. 1.**
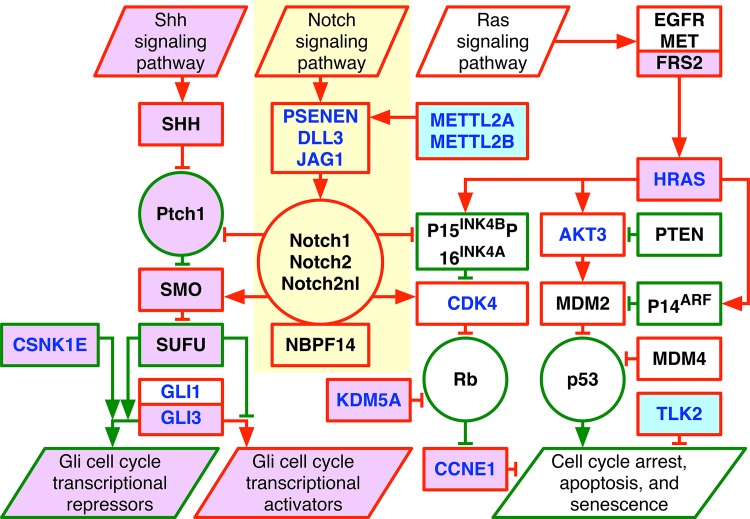
The genome-wide GBM pattern encodes human normal-to-tumor cell transformation via the Ras, Shh, and Notch pathways and includes most changes in chromosome numbers and focal CNAs that were known and at least as many that were unrecognized in GBM prior to its discovery. The genotype that the GBM pattern represents is depicted in a diagram of the WGS technology-filled in Notch pathway (yellow) in addition to the Agilent and Affymetrix microarray-described Ras and Shh pathways, including CNAs unrecognized in GBM prior to the discovery of the pattern (violet) and, among them, biochemically putative drug targets that are predicted to be correlated with survival (light blue). Shown are amplifications (red) and deletions (green) of genes and transcript variants (rectangles), either GBM- and LGA-shared (black) or GBM-specific (blue), and relationships that directly or indirectly lead to increased (arrows) or decreased (bars) activities of the genes and transcripts, the tumor suppressor proteins p53, Rb, and Ptch1, and the oncoproteins Notch1, Notch2, and the hominin-specific Notch2nl (circles).

Recently, a genome-wide pattern of co-occurring DNA copy-number alterations (CNAs) has been associated with a roughly one-year median survival in GBM patients,^24^ shorter than the standard life expectancy of roughly 15 months (Fig. 1). The pattern includes most changes in chromosome numbers and focal CNAs that were known in GBM prior to its discovery, most of which map to the rat sarcoma (Ras) signaling pathway.^25^ Also included are at least as many CNAs that were previously unrecognized in GBM, most of which map to the sonic hedgehog (Shh) and Notch signaling pathways that are known to induce the development of medulloblastoma and neuroblastoma brain cancers, respectively. Some of the naturally occurring alterations that are described by the pattern are analogous to the genetic elements that artificially transform human normal cells and their diploid nuclei into tumor cells with grossly polyploid nuclei.^26–28^

**FIG. 2.**
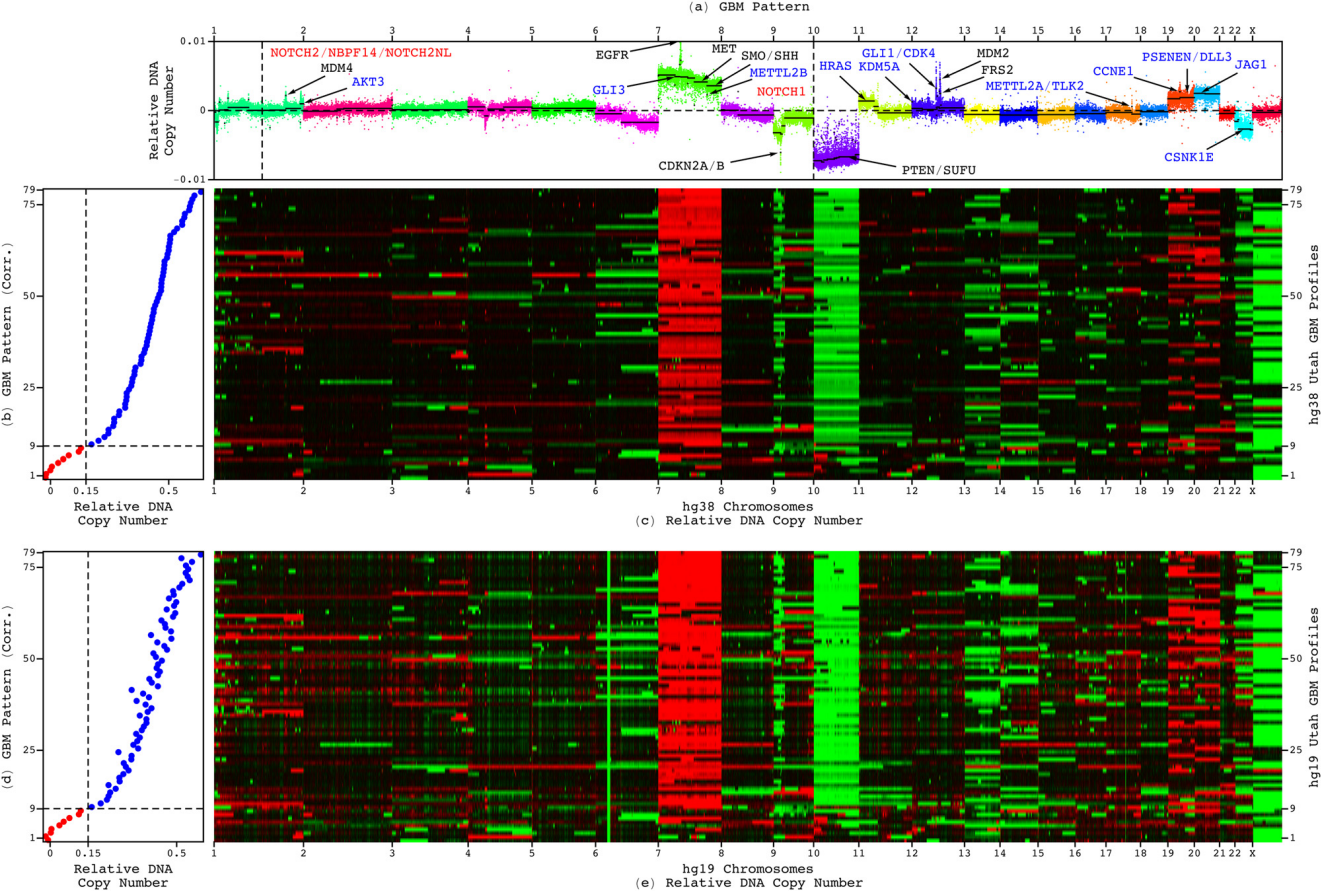
The classifications of the Utah set of 79 patients by the genome-wide GBM pattern are 100% consistent between the duplicate hg38 and hg19 whole-genome GBM profiles. (a) The GBM pattern, i.e., a genome-wide pattern of DNA CNAs, is displayed in a plot of relative copy numbers across the subset of 211 653 of the 212 696 Agilent probes that maintain the same chromosomal order in both reference human genomes, ordered and colored based upon their genomic coordinates, with the previously identified segments (black lines) and GBM-specific (blue), GBM- and LGA-shared (black), and WGS technology-filled in (red) CNAs. (b) The Pearson correlations of the hg38 profiles of the 79 patients with the GBM pattern are displayed in a plot, with the classification of the patients into low (red) or high (blue) correlations by using the previously established cutoff of 0.15. (c) The corresponding hg38 profiles are displayed in a raster of WGS read-count, i.e., DNA copy-number, amplification (red), no change (black), and deletion (green), from the medians of their autosomes excluding the outlying chromosomes 10 and 7 and the short arm of chromosome 9, i.e., 9p. (d) The correlations of the hg19 profiles, ordered by the correlations of the hg38 profiles, are displayed in a plot. (e) The corresponding hg19 profiles are displayed in a raster, showing the same genotype–phenotype relationship as the hg38 profiles.

**FIG. 3.**
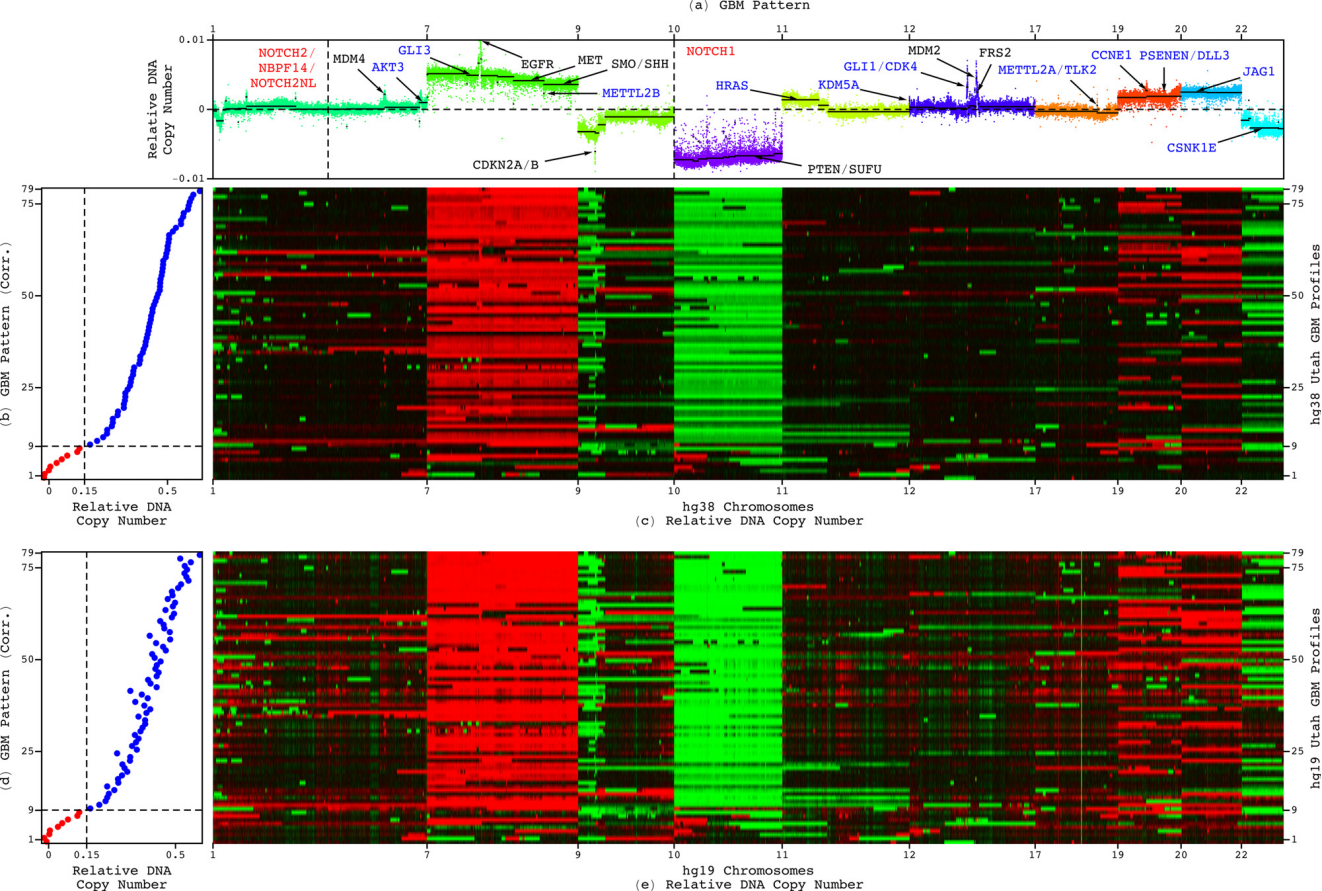
Notable in the GBM pattern, the deletion of chromosome 10, amplification of chromosome 7, and deletion of chromosome arm 9p, appear in the tumor genomes of some but not all 70 patients with high and, separately, some but not all nine patients with low correlations of their tumor profiles with the pattern. (a) The GBM pattern across the chromosomes 1, 7, 9, 10, 11, 12, 17, 19, 20, and 22, which are with notable CNAs in the pattern. (b) The correlations of the hg38 whole-genome GBM profiles with the genome-wide pattern. (c) The hg38 profiles across the chromosomes with notable CNAs in the pattern. (d) The correlations of the hg19 profiles with the pattern. (e) The hg19 profiles.

The genotype–phenotype relationship was discovered by using the non-domain-specific, i.e., universal, mathematical framework of the generalized singular value decomposition (GSVD)^29–31^ formulated as a comparative spectral decomposition.^32–35^ In the modeling of primary GBM tumor and patient-matched normal genomic profiles from the Cancer Genome Atlas (TCGA),^36^ the GSVD separated the tumor-exclusive relationship from those that are common to the tumor and normal genomes and from experimental batch effects. The genotype was represented by the GBM pattern across the tumor set of 212 696 Agilent comparative genomic hybridization (CGH) microarray probes mapped to the reference human genome hg18 (Figs. 2 and 3). The phenotype was established and validated by correlating the pattern with the tumor genomes of the mutually exclusive discovery and, separately, validation sets of 251 and 184 GBM patients, respectively. A non-negligible, i.e., high, correlation with the pattern conferred a shorter, roughly one-year, median survival also among the discovery and validation sets of 133 lower-grade, i.e., grades III and II, astrocytoma (LGA) patients and the discovery set of 85 GBM and LGA patients, profiled by Affymetrix single-nucleotide polymorphism (SNP) microarrays^37^ and whole-genome sequencing (WGS),^38^ respectively, and mapped to hg19.^39^

Note that primary GBM and LGA are different types of cancer. Their histopathologies overlap, but their epidemiologies differ.^40^ In the modeling of tumor and patient-matched normal profiles of the 59 and, separately, 85 patients of the Affymetrix LGA and WGS astrocytoma discovery sets, the GSVD separated an LGA pattern and an astrocytoma pattern across the tumor sets of ≈934K Affymetrix SNP probes and 2.8M WGS 1 K-nucelotide bins, respectively. The LGA pattern is encompassed by the GBM pattern, and the astrocytoma pattern is bounded by the GBM and LGA patterns.

By identifying the shorter survival phenotype among the TCGA patients, the GBM pattern predicted survival statistically better than and independent of the best other indicator, i.e., age, among the GBM patients and, in addition, grade among the GBM and LGA patients together. The pattern also predicted survival statistically better than and independent of the existing pathology laboratory tests, i.e., for *MGMT* promoter methylation and *IDH1* mutation, in general and in patients who received treatment, i.e., chemotherapy and radiation.

That the GBM pattern is a predictor independent of age, chemotherapy, and radiation implies that the information that the pattern contains is not currently being used in clinical practice. That the pattern is a predictor better than age additionally suggests that using the information can improve the prognostics, diagnostics, and therapeutics of the disease.

First, the prognostic classification by the pattern may help to manage GBM pseudoprogression for a few months after the start of chemotherapy and radiation, until it is possible to conclusively distinguish it from progression.^41^ Pseudoprogression presents with magnetic resonance imaging (MRI) features that mimic progression. Progression may necessitate immediate discontinuation of the treatment and intervention with a different regimen. In the case of pseudoprogression, however, the immediate discontinuation and intervention would be premature. A prognosis of longer survival may help to decide against immediate changes to the treatment and continued follow up by using MRI and other imaging modalities.^42^

Second, the diagnostic classification by the genome-wide GBM pattern may prove more relevant to identifying patients who would benefit from a drug than an assay of between one to a few hundred genes and genomic loci, even in the case of a drug that targets only one gene. This is because the activity of any one gene and the effect of targeting it depend upon the whole genome. For example, the amplification of the epidermal growth factor receptor-encoding gene (*EGFR*), which is notable in the pattern, is also frequently observed in GBM tumors. However, the effect of *EGFR* deficiency is known to depend upon the genetic background in which it occurs, i.e., the whole genome.^43^ It may not be surprising, therefore, that in a phase II clinical trial of an *EGFR* inhibitor, a patient's survival was independent of the tumor's *EGFR* amplification.^44^ By better identifying patients who would benefit from a drug, the pattern may help the drug progress from early tests to regulatory approval. Note that in 2003–2011, <7% of cancer drugs in general have advanced from phase I clinical trials to FDA approval, none of which are for GBM.^45^

Third, the therapeutic predictions of the pattern, that previously unrecognized, biochemically putative drug targets and combinations of targets are correlated with survival, may lead to new agents for treatment. These targets include, e.g., the transfer RNA (tRNA) methyltransferases-encoding genes methyltransferase-like 2A and 2B (*METTL2A/B*) and the serine/threonine kinase-encoding gene tousled-like kinase 2 (*TLK2*).^46,47^

Here, to test the genotype–phenotype relationship, we used an experimental workflow similar to that of TCGA and a computational workflow similar to that we previously used to establish and (re)validate the GBM pattern in the sets of GBM and LGA patients from TCGA (Methods Section and Fig. S1 in the supplementary material).

## RESULTS

### The Utah set of 79 patients statistically represents the U.S. adult GBM population

The distribution of the age at diagnosis of the 8001 Surveillance, Epidemiology, and End Results (SEER) patients between ≥50 years and <50 years, whose average is 63 years, is statistically indistinguishable from the distributions of the 79 Utah and 28 Case Western Reserve University (CWRU) patients, whose averages are 63 and 64 years, respectively, with the corresponding $\chi^{2}$ *P*-values >0.05 (Table S1). These distributions, however, describe statistically significantly older populations at diagnosis than that of the TCGA patients, whose average of 58 years reflects a bias against surgical resections in patients >65 years old. The cutoff of 50 years at diagnosis has been consistent with clinical observations since 1950 and was (re)established in clinical trials, e.g., in 1993 and 2008.

In Kaplan–Meier (KM) survival analyses,^48^ the approximately 11-month median survival times of the Utah and CWRU patients are statistically indistinguishable from the nine months of the SEER patients, with the corresponding log-rank *P*-values >0.05 (Fig. S2). These median survival times, however, are statistically significantly shorter than the 14-month median survival of the 443 TCGA patients, and reflect the differences in the age distributions, where an older age at diagnosis is associated with a shorter GBM survival time (Fig. S3).

We find, therefore, that the Utah set represents the U.S. GBM population and the CWRU surgical case series in terms of the disease phenotypes of age at diagnosis and median survival time and, similarly, also in terms of the normal phenotypes of sex, race, and ethnicity. By experimentally validating the genotype–phenotype relationship in the Utah set of 79 patients, we validate its applicability to the U.S. adult GBM population at large.

### Intratumor heterogeneity affects ≈11% and profiling technology and reference human genome specifics affect <1% of the classifications by the GBM pattern

The classifications of the 79 Utah patients and, similarly, the 18 Utah-TCGA patients, are 100% consistent between the duplicate hg38 and hg19 profiles. The experimental variation, in the aliquot DNA samples, the sequencing technologies, i.e., of the Broad Institute (BI) vs the Beijing Genomics Institute (BGI) -Shenzhen, and the reference human genomes, did not affect the classifications of the pairs of profiles of the same analyte DNA samples and, therefore, also the same tumor portions. The same DNA analyte samples and tumor portions of 231 of the 443 TCGA patients were profiled in either duplicates or triplicates by using a different profiling technology for each of the replicates from the three technologies of WGS and Affymetrix SNP and Agilent CGH microarrays. The previously computed classifications of the 231 patients are inconsistent between the two or among the three profiles of only two, i.e., <1%, of the 231 patients.

Note that the CGH and SNP microarrays together with WGS represent the main genomic profiling technologies, whereas the BI and BGI sequencing represent the main WGS technologies. Each technology relies on specific experimental designs and computational protocols, which are sensitive to changes, e.g., in the experimental batch or computational preprocessing. This has been shown to contribute to a low reproducibility, i.e., precision, of <70% in the classifications of normal copy-number variants (CNVs) of between one to a few hundred genes and genomic loci.^49^ In addition, there are disparities between the different reference human genomes that the profiles were mapped to by these technologies. For example, the short arm of chromosome 9, i.e., 9p, for which loss is associated with GBM, spans ≈51.8M, 49M, and 43M nucleotides in hg18, hg19, and hg38, respectively. Most of the 2.8M- and 6M-nucleotide updates, from hg18 to hg19, and from hg19 to hg38, map to the largest heterochromatin block in the human autosome, which encompasses the centromere of chromosome 9. The classifications by the genome-wide pattern, of hundreds of thousands of loci, however, are >99% precise, i.e., robust to changes in the genomic profiling technologies and the reference human genomes.

Of the 18 Utah-TCGA patients, the classifications of 16 patients are consistent between their Utah and TCGA profiles. The variation in the tumor samples and portions, i.e., of the Utah study vs TCGA, affected only two of the 18, i.e., ≈11% of the patients. Most batch differences except for intratumor heterogeneity^50^ between the Utah study and TCGA were controlled for by using an experimental workflow similar to that of TCGA and a computational workflow similar to that previously used to classify the sets of GBM and LGA patients from TCGA.

We find, therefore, that intratumor heterogeneity affects ≈11% of the classifications by the GBM pattern. In agreement with the previous modeling of genomic profiles from TCGA, profiling technology and reference human genome specifics affect <1% of the classifications. This reflects the increased reliability of measuring DNA vs less stable biomolecules, e.g., RNA, or biomolecules which abundance levels vary in time and space even within cells that share the same genome, e.g., RNA and proteins. This also reflects the decreased sensitivity of the pattern of hundreds of thousands of genes and genomic loci to the error in measuring any one of them vs that, e.g., of an assay of between one to a few hundred genes and genomic loci.

### The GBM pattern predicts survival better than and independent of age at diagnosis

**FIG. 4.**
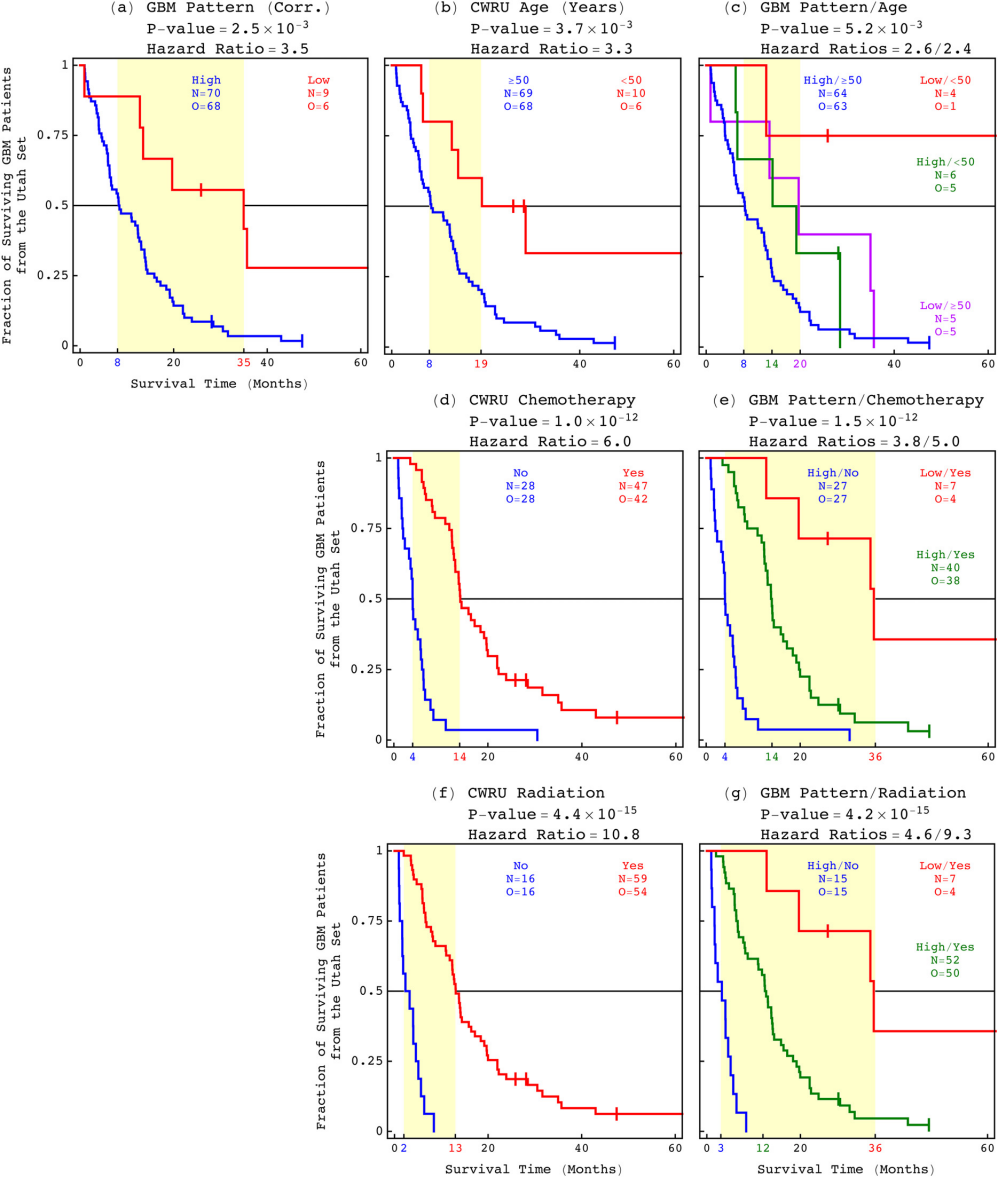
Among the 79 Utah patients, the GBM pattern predicts survival statistically better than the best other indicator, i.e., the patient's age at diagnosis. (a) The KM curves of the 79 patients classified by the GBM pattern are displayed in a graph, showing a 27-month, i.e., 2.25-year median survival difference (yellow) between the patients with high (blue) and low (red) correlations, respectively, with a corresponding log-rank *P*-value of 2.5 × 10^−3^ and a univariate Cox hazard ratio of 3.5. (b) The patients classified by age at diagnosis between ≥50 years and <50 years. (c) The 12-month median survival difference of the 69 patients who were ≥50 years at diagnosis, and are classified by the pattern, is at least comparable to the 11-month median survival of the classification of the 79 patients by age alone. (d) The median survival of the 47 patients who were treated with chemotherapy is 14 months. (e) A low correlation with the pattern identifies seven patients of a 36-month median survival among the 47 chemotherapy-treated patients. (f) The median survival of the 59 radiation-treated patients is 13 months. (g) A low correlation identifies the same seven patients of a 36-month median survival among the 59 radiation-treated as among the 47 chemotherapy-treated patients.

Of the Utah set of 79 patients, 70 patients are classified as having high, i.e., >0.15, and nine, i.e., roughly 10%, are classified as having low, i.e., <0.15 Pearson correlations of their tumor profiles with the GBM pattern (Fig. 4). These nine patients include the only two, i.e., ≈2.5%, of the 79 patients who were still alive 60 months, i.e., five years, from their diagnosis. In a KM survival analysis, the 70 patients with high correlations are of an approximately eight-month median survival, which is statistically significantly shorter than the 35 months of the nine patients with low correlations. The median survival difference is 27 months, i.e., 2.25 years, with the corresponding log-rank *P*-value of 2.5 × 10^−3^. In a univariate Cox proportional hazards model, a high correlation confers ≈3.5 times the hazard of a low correlation, with the corresponding Wald *P*-value of 4.3 × 10^−3^ (Table S2). The concordance index, i.e., accuracy, of the classification is ≈0.78, i.e., 78%. To compute the concordance index, all pairs of patients were counted, where each patient is from a different group of the KM analysis. Among these pairs, all pairs were counted where the observed ranking of survival between the patients agrees with the predicted ranking between the groups, based upon the median survival times estimated by the Cox model.

The KM median survival differences and univariate Cox hazard ratios of the 79 Utah patients and, separately, the 47 chemotherapy- and 59 radiation-treated patients among them, when classified by the GBM pattern, are greater than those when classified by age. The concordance indices of the 79, 47, and 59 patients, when classified by the pattern, are greater than or the same as those when classified by age. The log-rank and Wald *P*-values as well as the Akaike information criterion (AIC) values that correspond to the pattern are less than those that correspond to age. The bivariate hazard ratios of the classifications of the 79 patients by the pattern and age, and the 75 of the 79 patients with treatment information by the pattern and chemotherapy or radiation, are within the 95% confidence intervals of the corresponding univariate ratios.

**FIG. 5.**
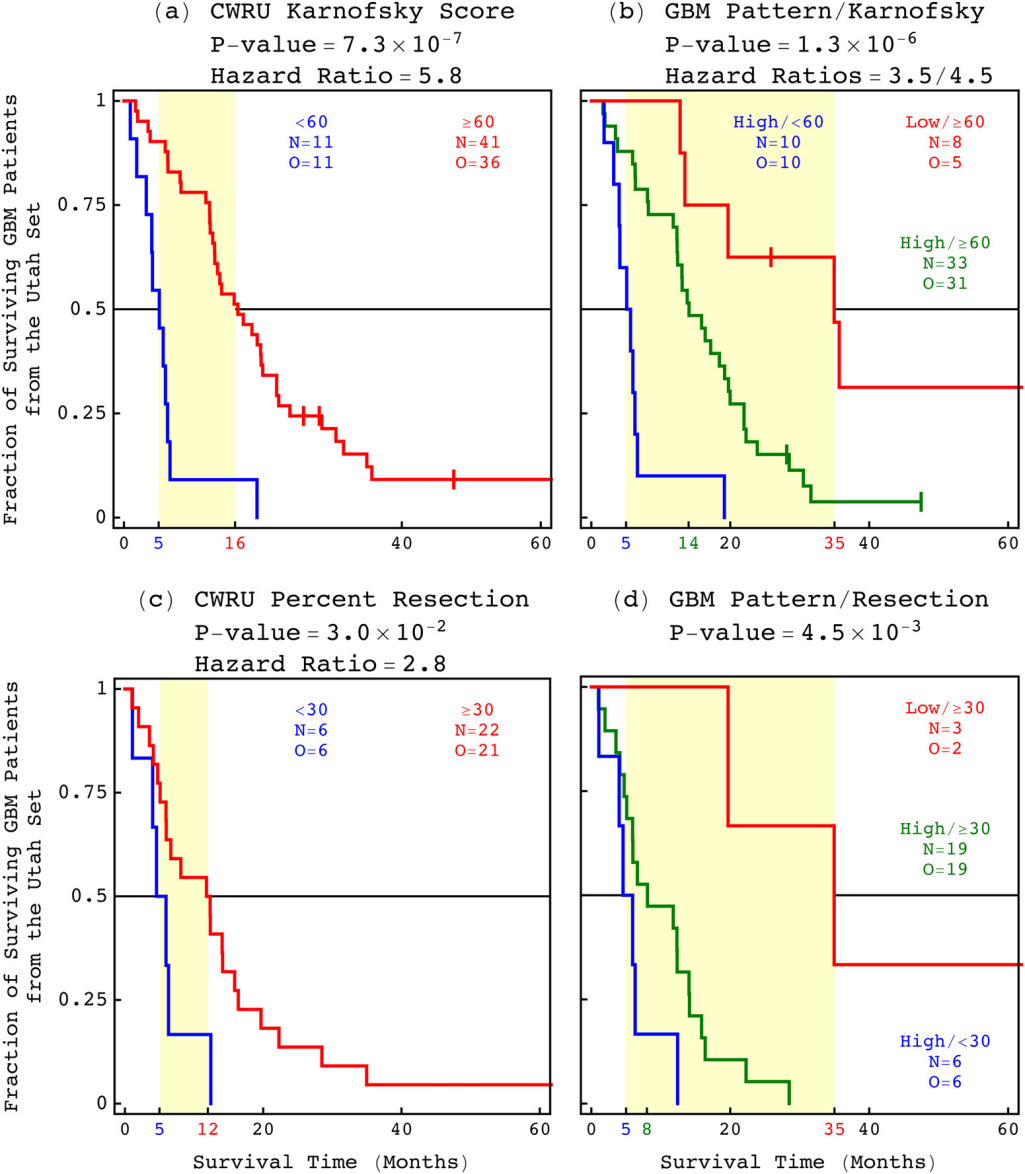
Among the Utah patients, the GBM pattern is a predictor of survival independent of the post-surgical resection metrics, i.e., the Karnofsky performance score and the percent primary tumor resection. (a) The median survival of the 41 patients with Karnofsky score ≥60 is 16 months. (b) A low correlation with the pattern identifies eight patients of a 35-month median survival among the 41 patients. (c) The median survival of the 22 patients with tumor resection ≥30% is 12 months. (d) A low correlation identifies three patients of a 35-month median survival among the 22 patients.

Similarly, the median survival differences of the 52 of the 79 patients with Karnofsky performance score information and, separately, the 28 of the 79 patients with percent primary tumor resection information, when classified by the GBM pattern, are greater than those when classified by the score and percent resection, respectively (Fig. 5). The univariate ratios of the classification by the pattern are within the 95% confidence interval of the corresponding bivariate ratios of the classification by the pattern and the score or percent resection. The cutoff of a Karnofsky score of 60 is consistent with the score of 70 established in clinical trials, when taking into account the TCGA standard intervals of 20. A recent study suggested 70% primary tumor resection as the lowest cutoff in increments of 5% to confer survival advantage.^51^ The range of resection among the 28 of the 79 Utah patients, however, is 7%–69%. We, therefore, used the cutoff of 30% primary tumor resection, which is the lowest cutoff in increments of 5% to confer survival advantage among the 28 patients.

Note that experimental, biological, and clinical parameters other than survival are statistically indistinguishable in the classification of the 79 patients by the pattern, with the corresponding Mann–Whitney–Wilcoxon (MWW) *P*-values >0.05 (Fig. S4). These parameters include, e.g., the patient's diagnosis year, the tumor sample's weight, the slide's percent tumor cells, the analyte and aliquot DNA samples' weights, and the hg38 and hg19 WGS profiles' coverages.

We find, therefore, in agreement with our previous mathematical modeling of genomic profiles from TCGA, that the correlation of a tumor's whole genome with the GBM pattern predicts survival statistically better than and independent of the best other indicator, i.e., the patient's age at diagnosis. Combined with age, the pattern is an even better predictor of survival. This is in general as well as in patients who receive treatments, i.e., chemotherapy and radiation. The pattern is a predictor of survival independent of chemotherapy and radiation and the post-surgical resection metrics, i.e., the Karnofsky performance score and the percent primary tumor resection.

The classifications by the existing pathology laboratory tests are statistically insignificant for the 79 Utah patients, where only eight and 46 patients have *MGMT* promoter methylation and *IDH1* mutation test results, respectively (Table S3). In addition, the testing assays were not standardized. The distributions of the test results of the Utah patients, however, are indistinguishable from the distributions of the 443 TCGA and 28 CWRU patients for whom *MGMT*^52^ and *IDH1* were consistently evaluated, with the corresponding $\chi^{2}$ *P*-values >0.05.

The univariate Cox hazard ratios of the 255 and, separately, 329 of the 443 TCGA patients with *MGMT* and *IDH1* test results, when classified by the GBM pattern, are greater than or the same as those when classified by *MGMT* and *IDH1*, respectively (Fig. S5 and Table S4). The log-rank and Wald *P*-values as well as the AIC values that correspond to the classifications by the pattern are less than those that correspond to the classifications by *MGMT* and *IDH1*. The bivariate hazard ratios of the classifications of the 255 and 329 patients by the pattern and *MGMT* or *IDH1*, respectively, are within the 95% confidence intervals of the corresponding univariate ratios. Consistently, e.g., among the nine Utah patients that are classified as having low correlations with the pattern, *IDH1* mutation was not detected in two of the five patients with test results. Among the 70 Utah patients that are classified as having high correlations, *IDH1* mutation was detected in two of the 41 patients with results.

Similarly, the KM median survival difference, univariate Cox hazard ratio, and concordance index of the 107 TCGA patients with *TERT* mRNA expression information,^53^ when classified by the GBM pattern, are greater than those when classified by *TERT* (Fig. S6). The median survival times of the 407 TCGA patients with a proneural, mesenchymal, classical, or neural GBM subtype designation, which is based upon the expression of a few hundred genes, when classified into these subtypes, are statistically indistinguishable, with the corresponding log-rank *P*-value >0.05 (Fig. S7).

As in the classification of the Utah patients, the bivariate hazard ratios of the 335 of the 443 TCGA patients with Karnofsky performance score information, when classified by the pattern and the score, are within the 95% confidence intervals of the corresponding univariate ratios (Fig. S8). Parameters other than survival are indistinguishable in the classification of the TCGA patients by the pattern, with the corresponding MWW *P*-values >0.05 (Fig. S9).

The distributions of the GBM pattern of the 79 Utah, 443 TCGA, and 28 CWRU patients are indistinguishable. Like the Utah patients, roughly 10% of the TCGA and CWRU patients are classified as having low correlations of their tumor profiles with the GBM pattern. These include five of the 12, i.e., ≈2.7%, of the 443 TCGA patients who were still alive five years from their diagnosis. The CWRU patients additionally represent the Utah patients in terms of the disease phenotype of median survival conferred by a high correlation with the pattern. Among the CWRU patients, a high correlation confers a shorter, 11-month, i.e., roughly one-year median survival time, which is indistinguishable from the approximately eight-month median survival of the Utah patients, with the corresponding log-rank *P*-value >0.05 (Fig. S10).

We find, therefore, that the TCGA patients and the CWRU surgical case series statistically represent the Utah patients in terms of the disease phenotypes of *MGMT* promoter methylation, *IDH1* mutation, as well as the GBM pattern. Among the TCGA patients, the pattern predicts survival better than and independent of the existing pathology laboratory tests, i.e., for *MGMT* promoter methylation and *IDH1* mutation, as well as better than *TERT* gene expression, the most recent indicator of survival to have advanced to GBM standard of care. Combined with either test, for *MGMT* or *IDH1*, the pattern is an even better predictor of survival.

## DISCUSSION

We have experimentally validated a clinically actionable genotype–phenotype relationship, where a non-negligible, i.e., high, correlation of a primary GBM tumor's genomic profile with a genome-wide pattern of co-occurring DNA CNAs confers a patient's shorter, roughly one-year, median survival. That the GBM pattern recurs in, i.e., has a high correlation with roughly 90% of the Utah and, separately, TCGA tumor profiles, suggests that it is selected for by the evolutionary forces that affect the brain cancer development. A randomly occurring variation of copy numbers across the N≫1 genomic loci is N−1≫1 times more probable to have a low correlation with the pattern. It is possible, therefore, that a low correlation with the pattern reflects a detection of the tumor somewhat early in its development.

This genotype–phenotype relationship can improve the prognostics, diagnostics, and therapeutics of GBM, which remained largely unchanged for decades. The prognostic classification by the pattern may help to manage pseudoprogression after the start of treatment, i.e., chemotherapy and radiation. The diagnostic classification may help drugs progress from early tests to regulatory approval. The therapeutic predictions, of previously unrecognized, biochemically putative drug targets and combinations of targets that are correlated with survival, may lead to new agents for treatment.

### A GBM patient's survival is the outcome of their tumor's whole genome

The pattern includes most CNAs that were known to recur in subsets of GBM tumors and at least as many that were unrecognized in GBM prior to its discovery. Together, but not separately, these co-occurring CNAs encode for human normal-to-tumor cell transformation, including, e.g., polyploidy, via the Ras, Shh, and Notch developmental and growth signaling pathways.

For example, the deletion of chromosome 10, the amplification of chromosome 7, and the deletion of the chromosome arm 9p, which are notable in the pattern, are also frequently observed in GBM tumors. Repeated previous attempts, however, to associate these chromosome number changes alone with survival were unsuccessful. Similarly, in our previous mathematical modeling of genomic profiles from TCGA, where our classification of chromosome number changes was confirmed by a 100% match between the sex of the patients and the numbers of X chromosomes in their normal genomes, neither one nor any combination of these chromosome number changes in the tumor genomes was correlated with survival (Fig. S11).

**FIG. 6.**
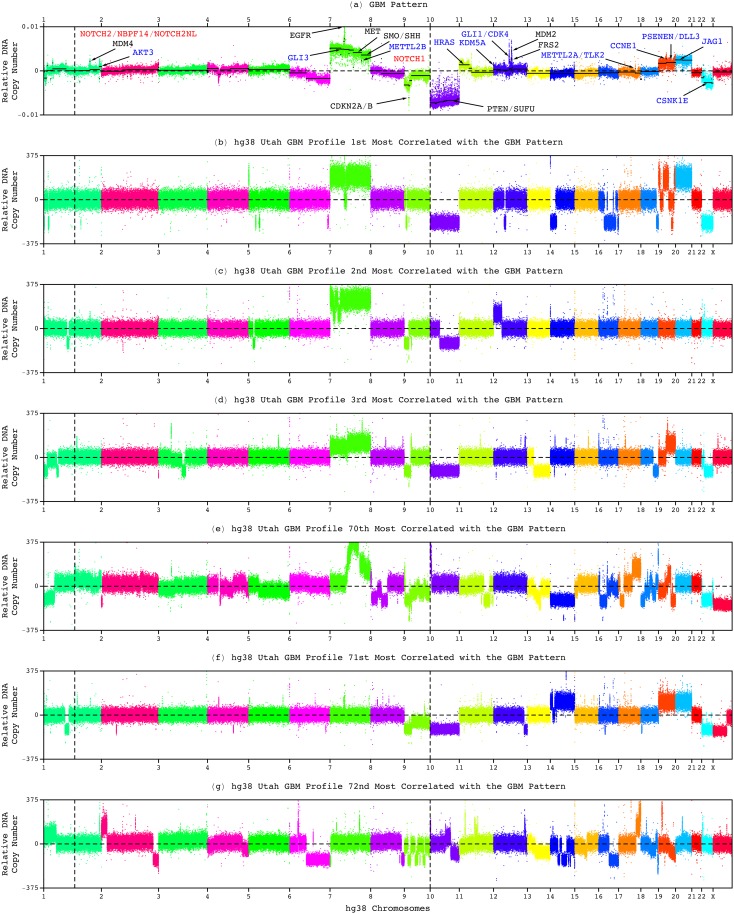
Frequently observed in GBM tumors and notable in the GBM pattern, neither one nor any combination of chromosome 10 deletion, chromosome 7 amplification, and chromosome arm 9p deletion, distinguishes hg38 tumor profiles that have a high correlation with the pattern from those that have a low correlation. (a) The genome-wide pattern and the hg38 whole-genome profiles that are (b) first, (c) second, (d) third, (e) 70th, (f) 71st, and (g) 72nd most correlated with the pattern.

**FIG. 7.**
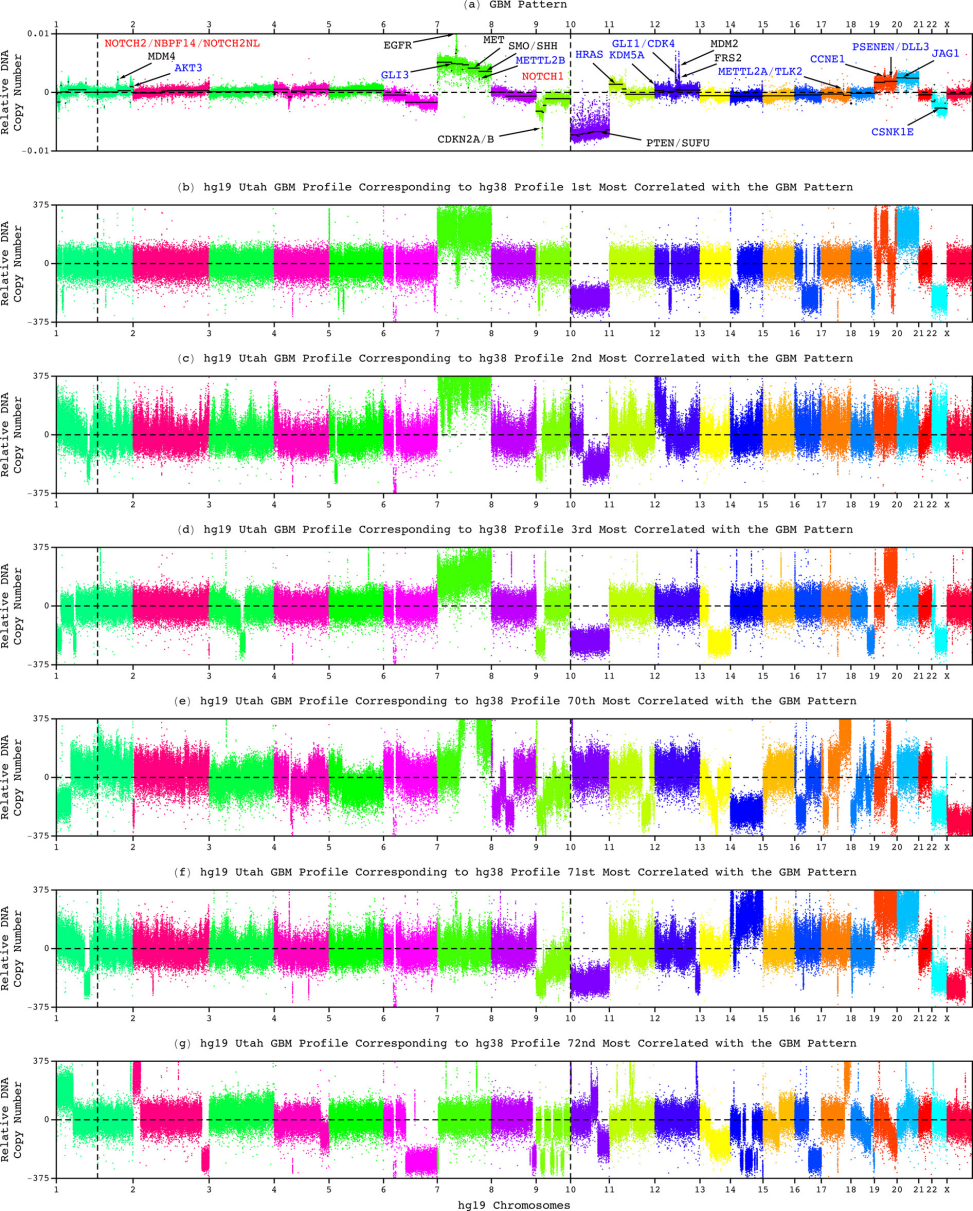
Neither one nor any combination of chromosome 10 deletion, chromosome 7 amplification, and chromosome arm 9p deletion, distinguishes hg19 tumor profiles that have a high correlation with the GBM pattern from those that have a low correlation. (a) The genome-wide pattern and the hg19 whole-genome profiles corresponding to the hg38 profiles that are (b) first, (c) second, (d) third, (e) 70th, (f) 71st, and (g) 72nd most correlated with the pattern.

Here, among the Utah patients, these chromosome number changes appear in the tumor genomes of some but not all of the 70 patients with a shorter, roughly one-year, median survival as well as, separately, the nine patients with a longer median survival (Figs. 6 and 7). While the whole of chromosome 10 appears deleted in the tumor genomes with the hg38 profiles that are, e.g., the first, third, and 71st most correlated with the GBM pattern, with the corresponding correlations of ≈0.64, 0.60 > 0.15, and 0.13 < 0.15, respectively, only a partial deletion appears in the tumors with the profiles that are, e.g., second and 72nd most correlated, with the correlations of 0.61 > 0.15 and 0.12 < 0.15, and no alteration appears in the tumor with the profile that is, e.g., the 70th most correlated, with the correlation of 0.17 > 0.15.

In another example, among the TCGA patients, log-rank test *P*-values that are statistically significant, i.e., <0.05, were computed for only 12 of the 130 segments identified in the GBM pattern by circular binary segmentation.^54^ The KM median survival time difference computed for an alteration in either one of these 12 segments is at most ≈11 months, i.e., roughly two thirds of the 16 months observed in the classification of the patients by the pattern. Median survival differences of at least five months, i.e., roughly a third of the 16 months in the classification by the pattern, were computed for only six of the 12 segments. Two of these six segments include biochemically putative drug targets that were previously unrecognized in GBM (Fig. S12). One segment encompasses the tRNA methyltransferase-encoding *METTL2B*. The other encompasses the *METTL2B*-homologous gene *METTL2A* and the nuclear localization sequence-encoding part of the serine/threonine kinase-encoding *TLK2*.^55^

We conclude that a GBM patient's survival is the outcome of their tumor's co-occurring, possibly coordinated, DNA CNAs across the whole genome.

### Proof of principle that comparative spectral decompositions are uniquely suitable for discovering clinically actionable, accurate, and precise, genotype–phenotype relationships

That the prognostics, diagnostics, and therapeutics of GBM remained largely unchanged for decades illustrates the challenges of discovering genotype–phenotype relationships in cancer and other disorders in general. This is especially so for DNA copy-number genotypes. Copy-number changes overlap ≈12% of the normal human genome, where they are 10^2^–10^4^ times more frequent than point mutations,^56^ and are implicated in both normal and tumor development.^57^ Yet, to date, efforts to link disease susceptibility with CNVs vs those with, e.g., SNPs, yielded less than one CNV association to each 50 SNP associations.^58^ Similarly, repeated previous attempts to associate DNA CNAs with GBM survival were unsuccessful.

The GBM pattern was only recently discovered, and only by using the GSVD formulated as a comparative spectral decomposition. A chromosome arm-wide pattern of co-occurring DNA CNAs that has been associated with shorter survival in lung, ovarian, and uterine adenocarcinomas was only recently discovered, and only by using the GSVD and the tensor GSVD, which is another comparative spectral decomposition.^59,60^ We defined the comparative spectral decompositions to simultaneously identify the similarities and dissimilarities among multiple datasets recording different aspects of interrelated phenomena.

For example, the GSVD separates any two datasets that record patient-matched tumor CNAs and normal CNVs into pairs of combinations of variations. In each pair, the variation across the set of patients is shared by both the tumor and normal combinations. In addition, the tumor combination includes a variation across the tumor genomic loci, and the normal combination includes a variation across the normal genomic loci, which, in general, are different. Each pair of combinations corresponds to a pair of weights, i.e., superposition coefficients, one in the tumor dataset and the other in the normal dataset. When neither weight is negligible relative to the other, both the normal and tumor combinations are interpreted to represent a normal genotype–phenotype relationship that is conserved in the tumor, e.g., a pattern of X chromosome variation relative to the autosome in both the normal and GBM tumor genomes has been associated with the sex of the patients. When the weight in the normal dataset is negligible relative to the weight in the tumor dataset, the tumor combination is interpreted to represent a tumor-exclusive genotype–phenotype relationship, e.g., the genome-wide GBM pattern of DNA CNAs in the tumor genomes has been associated with the survival of the patients. Here, we experimentally validated this relationship, which is statistically more accurate and more precise than, as well as independent of, the best GBM indicators and tests in clinical use.

The unsupervised, i.e., data-driven, GSVD and other comparative spectral decompositions in general, are sensitive to accurate and precise genotype–phenotype relationships that other machine learning methods miss. This includes small discovery sets of only, e.g., 251 and 85 patients, and imbalanced validation sets of, e.g., 184 and 79 patients, with large tumor and normal genomic profiles of, e.g., roughly 213 K microarray probes and 2.8M WGS bins each. This is because comparative spectral decompositions, which scale to petabyte-sized datasets and beyond, use the complex structure of the datasets, of patient-matched minimally preprocessed genome-scale profiles, rather than simplifying the datasets and standardizing the profiles based upon assumptions as is commonly done.

We conclude that comparative spectral decompositions underlie a non-domain-specific, i.e., universal mathematical description of the genotype–phenotype relationships in cancer that other machine learning methods miss. These genome-scale relationships are clinically actionable and can improve the prognostics, diagnostics, and therapeutics of the disease. Like the modeling of normal and patient-matched tumor genomes can discover relationships relevant to cancer, the modeling of normal and patient-matched viral genomes or genomes of microbiomes can, respectively, inform personalized infectious disease medicine or personalized health in general.

## METHODS

Tumor samples were collected at the CWRU TCGA tissue source site from adult patients diagnosed with primary GBM undergoing surgical resection between 2007 and 2017, who were retrospectively enrolled in this study (Dataset S1). DNA was extracted from the GBM tumor samples at the Nationwide Children's Hospital (NCH) biospecimen core resource. The DNA was WGS-profiled in duplicates at a genomic characterization center, viz., BI, and BGI-Shenzhen, and the profiles were mapped to hg38 and hg19, respectively. The WGS compressed alignment map (CRAM) and binary alignment map (BAM) files, from BI and BGI, respectively, were minimally preprocessed at the University of Utah. The total size of the raw BAM and CRAM files was ≈13 terabytes or 0.01 petabytes. This resulted in 97 hg38 and 97 hg19 read-count profiles from the tumors of 97 patients (Supplementary Methods in supplementary material).

### Classification of the tumor profiles by correlation with the GBM pattern

To classify the hg38 and, separately, hg19 tumor profiles of the 97 patients, the Pearson correlation was computed between each tumor profile and the GBM pattern. The cutoff of 0.15 was used to separate profiles of negligible, i.e., low, correlation with the pattern from profiles of non-negligible, i.e., high, correlation. This cutoff was previously established for the Agilent GBM discovery set of TCGA patients and (re)validated for the Agilent GBM validation, Affymetrix LGA discovery and validation, and WGS astrocytoma discovery sets of patients.

The mathematical range of a Pearson correlation is ±1. The range of the correlations computed for the 248 of the 251 patients of the Agilent GBM discovery set that are with clinical information in TCGA is ≈[−0.12,0.81]. Of the 184 patients of the Agilent GBM validation set, the correlations computed for 183 are in the range of [−0.14,0.77], where the correlation computed for the remaining patient is –0.16. Here, the ranges of correlations computed for the hg38 and hg19 tumor profiles of the 97 Utah patients are ≈[−0.02,0.64] and [−0.02,0.60], respectively. The cutoff of 0.15, therefore, separates non-negligible correlations in the range of >0.15 from negligible correlations that range from <0.15 to roughly –0.15.

The Pearson correlation is a measure of the similarity between the GBM pattern and a tumor profile in terms of their variations from their means and relative to their standard deviations. At the mathematical upper and lower bounds, the correlations of ±1 correspond to profiles that vary from their means and relative to their standard deviations either exactly as or exactly opposite to the pattern. A correlation of 0 corresponds to any profile, which variation is orthogonal to, i.e., independent of, the pattern. Across N≫1 genomic probes or bins, there mathematically exist N−1≫1 independent variations that are exactly orthogonal to the pattern as well as to each other. The correlation estimates the weight, i.e., superposition coefficient, of the GBM pattern in the tumor profile, relative to the weights of these independent variations that are exactly orthogonal to the pattern.

It is not surprising, therefore, that while the tumor profiles with non-negligible, i.e., high, correlations with the GBM pattern are roughly similar to each other in addition to the pattern in terms of their variations across the genome, the profiles with negligible, i.e., low, correlations are different from each other as well as from the pattern.

### Construction of the Utah, Utah-TCGA, TCGA, CWRU, and SEER sets of patients

The 97 patients were separated into the Utah-TCGA set of 18 patients, which data were included in the previous modeling of genomic profiles from TCGA, and the Utah set of 79 patients, which data were not previously modeled. For comparison with the previous modeling, a TCGA set of 443 patients was constructed (Dataset S2). This set combines the 248 of the 251 patients of the Agilent GBM discovery set that are with clinical information in TCGA, the 184 patients of the Agilent GBM validation set, and the 11 GBM patients of the WGS astrocytoma discovery set that are exclusive of both Agilent sets. For comparison with the CWRU surgical case series, a CWRU set of 28 patients was constructed. This set corresponds to those among the TCGA set of 443 patients that enrolled in TCGA via CWRU. The previously computed classifications were used for both the TCGA and CWRU sets. Note that the Utah set of 79 patients is exclusive of both the TCGA and CWRU sets.

For comparison with the U.S. GBM population, a SEER set of 8001 patients was constructed from the SEER 2019 release covering 1975–2016 (Dataset S3). Adult patients diagnosed with primary GBM in 2007–2016 were selected to approximately match the Utah set, and from the nine registries that contributed to SEER continuously from 1989 to approximately match the TCGA set, which patients were diagnosed between 1989 and 2010. Patients were excluded who had more than one primary tumor or were missing dates of diagnosis and last contact.

## ETHICS APPROVAL

The Institutional Review Board (IRB) at the University Hospitals of Cleveland (CASE 1307-CC296, Ohio Brain Tumor Study) approved the use of de-identified clinical data and biospecimens in this Utah study.

## SUPPLEMENTARY MATERIAL

See the supplementary material for Supplementary Methods, Figs. S1–S12, Tables S1–S4, and Datasets S1–S3, also available at https://alterlab.org/GBM_retrospective_clinical_trial/.
